# Pharmacological modulation of directed network communication and neural hubs in action–effect integration

**DOI:** 10.1093/ijnp/pyaf031

**Published:** 2025-05-27

**Authors:** Jasmin Mayer, Anna Helin Koyun, Moritz Mückschel, Veit Roessner, Bernhard Hommel, Christian Beste

**Affiliations:** Cognitive Neurophysiology, Department of Child and Adolescent Psychiatry, Faculty of Medicine, TU Dresden, Dresden, Germany; Cognitive Neurophysiology, Department of Child and Adolescent Psychiatry, Faculty of Medicine, TU Dresden, Dresden, Germany; Cognitive Neurophysiology, Department of Child and Adolescent Psychiatry, Faculty of Medicine, TU Dresden, Dresden, Germany; Cognitive Neurophysiology, Department of Child and Adolescent Psychiatry, Faculty of Medicine, TU Dresden, Dresden, Germany; German Center for Child and Adolescent Health (DZKJ), Partner Site Leipzig/Dresden, Dresden, Germany; School of Psychology, Shandong Normal University, Jinan, China; Cognitive Neurophysiology, Department of Child and Adolescent Psychiatry, Faculty of Medicine, TU Dresden, Dresden, Germany; German Center for Child and Adolescent Health (DZKJ), Partner Site Leipzig/Dresden, Dresden, Germany; School of Psychology, Shandong Normal University, Jinan, China

**Keywords:** intentional action, action control, theta, catecholamines, methylphenidate

## Abstract

**Background:**

Acting intentionally requires individuals to anticipate the effects of their actions. Recent work has revealed the neural oscillatory dynamics underlying the establishment of action–effect bindings, which are vital to anticipating action effects. However, the neurobiological basis of these processes is elusive.

**Methods:**

Healthy adult participants (*N* = 54) engaged in a double-blind, counter-balanced, placebo-controlled experiment in which they worked in an experiment able to examine how action effects are planned, anticipated, and processed under placebo and methylphenidate conditions. Electroencephalogram data were analyzed to investigate the directed communication in cortical networks underlying action effect integration.

**Results:**

We show that an increase in catecholaminergic system activity alters the strength of directed communication in a cortical theta frequency network constituted by the insular cortex, the anterior temporal lobe, and the inferior frontal cortex. Additionally, pharmacological modulation regulates which of the brain structures act as a hub in different phases of the action–effect binding process.

**Conclusions:**

The findings highlight how the neural organization of processes supporting intentional action can be optimized neurobiologically through the catecholaminergic system.

**Significance Statement**

The catecholaminergic system plays a role in many neuropsychiatric disorders in which alterations in purposeful actions are reported (incl. Attention-deficit/hyperactivity disorder). Pharmacological modulations of this system (eg, through methylphenidate) play a major role in therapies for such disorders. Here, we show that the catecholaminergic system is likely key to understanding the neural processes underlying the acquisition of purposeful actions. We show how modulating catecholamines dynamically reconfigure brain networks underlying purposeful actions. The results provide insights into how purposeful actions are built with relevance to the understanding of neuropsychiatric disorders.

## INTRODUCTION

Acting intentionally is a key ability of humans, and various cognitive science concepts deal with how intentional actions can be carried out. According to ideomotor theorizing,^1,2^ the anticipation of a (sensory) action effect is central to developing the ability to act intentionally.^3-5^ There is ample evidence that people integrate representations of the sensory effects of an action and the motor pattern that produces these effects into enduring action–effect bindings (AEBs), which represent the building blocks of intentional actions. Once built, stored AEBs—so-called action–effect representations—are then reactivated, either by the active anticipation of intended action effects^5^ or by external stimuli resembling action effects,^3^ which then spread activation to the integrated motor patterns to carry out an intentional act.

While functional imaging has provided insights into the neural substrates of AEBs,^5-8^ understanding the spatiotemporal dynamics—how these representations unfold in time and space—has only recently gained attention through studies on oscillatory activity^9,10^ and cognitive functions.^11^

Notably, theta-band activity has emerged as particularly relevant for the formation and management of AEBs, aligning with broader findings that theta oscillations play a crucial role in coordinating perception–action representations.^12-16^ Importantly, AEBs involve a dynamic process: The integration of motor actions with their sensory outcomes into retrievable associations (action effect representation) that inform future action selection, emphasizing the need to understand when and how these bindings are established and retrieved. This distinction is crucial for understanding how different phases of AEB establishment and retrieval can be differentiated, offering a more mechanistic perspective on action–effect learning rather than simply describing general cognitive functions. Given the rapid temporal dynamics involved in AEB processing, electrophysiological methods such as electroencephalogram (EEG)—which offer superior temporal resolution—are more appropriate than functional magnetic resonance imaging (fMRI) for capturing the fine-grained neural communication underlying these processes. Moreover, the conceptual account of event file binding as the outcome of AEBs stresses the specific role of distinct fractions of neural activity (ie, theta-band activity),^12^ which can only be examined electrophysiologically.

Through the same electrophysiological approach as applied in the current study, a specific network of cortical regions has been implicated in AEB processing^10^: The anterior temporal lobe (ATL) plays a key role in theta-band neural dynamics and appears to integrate actions and their effects, receiving input from both the insular cortex (IC) and inferior frontal cortex (IFC).^10^ This input may be relevant for the control of intentional (ideomotor) action because the IC seems to link perception and action,^10^ while IFC seems to exert control over the ATL during the perception of anticipated action effects, with the ATL retrieving the predicted action–effect representation from the IFC.^10^ Although this IC–ATL–IFC network has been identified in previous studies,^10^ the neurobiological mechanisms driving this network are unclear. Clarifying these mechanisms is critical, as they determine how efficiently AEBs are formed and accessed. The available evidence shows that the strength of perception–action representations is increased when amplifying the dopaminergic and norepinephrinergic system using methylphenidate (MPH)^17,18^—a combined dopamine and norepinephrine transporter blocker.^19^

In this study, we thus used MPH as a pharmacological tool to probe the dependence of the AEBs on the catecholaminergic system, and we did so for the following reasons: Increasing dopamine and norepinephrine concentrations has effects on basic aspects of information processing that have been conceptualized as gain modulation principles.^20,21^ Gain modulation principles have often been discussed to affect the signal-to-noise ratio within neural circuits,^22-24^ but they can also affect network communication—that is, how information is routed through different network components.^24-26^ Gain modulation is of particular relevance when it comes to perceptual processes,^27^ and it has been shown that it modulated how event files are handled.^17^ Since AEB very much relies upon the integration of perceptual consequences of actions and gives rise to the building of event files, gaining control is of relevance to AEBs. Importantly, gain modulation can manifest in networked communication. Since directed communication IC–ATL–IFC network is central for AEB,^10^ MPH-induced modulations of gain control could lead to changes in directed connectivity.^28,29^ Our focus was not merely on whether MPH enhances perception–action integration but on how it reorganizes the directional connectivity within this specific neural circuit across the different phases of AEB processes. We hypothesized that MPH affects the directed communication in the IC–ATL–FC network underlying AEBs in general and the directionality of this communication in particular.^10^

To test this, we used an EEG frequency tagging approach to monitor for the activation of action–effect representations over time.^10^ This approach leverages the brain’s tendency to represent stimuli presented at a particular frequency (eg, flicker stimuli) through neural processes that exhibit the same frequency. This allows us to “frequency-tag” the processes that are triggered by a particular stimulus. Once the participants had acquired these action effects, they showed increased brain activity in the frequency with which the to-be-generated action effect was previously presented. This provides evidence that people indeed anticipate intended action effects before acting. However, our primary interest was not the frequency tagging itself but the directed connectivity underlying processes of the building of AEBs. By applying EEG-based directed connectivity analysis based on nCREANN (nonlinear Causal Relationship Estimation by Artificial Neural Network),^30^ we were able to trace not only where in the brain action–effect integration occurred but also how information spread across regions, especially during the transition from encoding to retrieval. This approach allowed us to investigate the mechanistic impact of MPH on AEB and retrieval processes. Moreover, following this approach, we could determine the neural communication pathways of AEBs in the course of action control. That is, we were specifically interested in the directed network communication involved in the AEB and retrieval processes and the modulatory effects of MPH.

## MATERIALS AND METHODS

Full experimental details are given in the Supplemental Material.

### Sample and MPH Administration

In total, 64 healthy participants were recruited and participated in this study. Eight participants were excluded because of technical issues during the data collection, and one participant dropped out. The sample entering the analysis consisted of *N* = 54 participants (25 females, 28 males; mean age 24.7 ± 2.5 years). One participant was excluded from connectivity and time–frequency (TF) wavelet analysis due to excessive artifacts (*n* = 53). Participants received a monetary expense allowance or course credit. All participants were tested in 2 sessions in a cross-over study design. During 1 session, participants were orally administered methylphenidate (MPH; 0.25 mg/kg, on average 18.7 mg), while in the other session, they received a placebo. The order in which participants received MPH or the placebo was pseudorandomized, considering biological sex and task condition. Female participants were only included if they were taking contraceptives. All participants were screened with self-report psychometric questionnaires for the following criteria: no psychiatric and neurological disorders in the past or present, no history of cardiovascular disease, self-reported alcohol use below 16 drinks per week, no use of psychoactive medication, and no history of brain surgery. Participants received written and oral information about the potential side effects of MPH. All participants provided informed written consent for study participation. The study was conducted as a double-blind study; the participants were debriefed after the second session. The study received ethical approval from the Ethics Committee of the TU Dresden.

### Task

The study used a recently developed forced-choice task^10^ to investigate action–effect associations. Action–effect associations were established by pairing a specific keypress with a visual stimulus and a specific sound (action effect). The visual stimulus consisted of flickering images of a cartoon-style sheep or a cat (flicker frequency of 4.5 or 8 Hz), the corresponding sound was a cat or sheep call presented via headphones at 55 dB. At the beginning of each trial, participants were instructed to focus on a gray circle of 1 cm radius that was presented at the center of the screen. After 2000 ms, the color of the circle switched to blue or red (cue stimulus). This colored circle was displayed for 2000 ms. Participants were instructed to press a key (either the right or left CTRL key) based on the dot’s color, following the stimulus offset and within a response window of 1000 ms. Correct responses triggered the immediate presentation of the visual and acoustic action effect for 2000 ms. No action effect was presented if the response was incorrect, too late, or missing. The stimulus-key pairings were randomized across participants to ensure equal distribution, resulting in 16 unique experimental conditions of cue (red/blue), response (left/right), action effect (cat/sheep), flicker frequency (4.5/8 Hz). The conditions were held constant per participant so that the participants conducted the same task in both sessions. The first session started with 30 practice trials, ensuring action–effect associations were successfully acquired prior to the main experiment trials. The experiment itself consisted of 300 randomized trials, 150 trials of each flicker frequency. The experiment was performed using Presentation software (Version 18.3, Neurobehavioral Systems, Inc., Berkeley, CA, www.neurobs.com). Participants were seated in front of a 24-inch TFT.

### EEG Recording and Preprocessing

EEG recordings were done with QuickAmp and BrainAmp amplifiers (Brain Products GmbH, Gilching, Germany), applying 60 Ag–AgCl electrodes in an equidistant setup with a 500 Hz sampling rate (reference electrode at Fpz, impedances <5 kΩ). Preprocessing was done in Automagic^31^ using EEGLAB^32^ and Matlab (2021b, The MathWorks Corp). After downsampling to 256 Hz, the EEGLAB clean_rawdata and PREP pipeline was applied, including line noise elimination, bad channel removal and artifact correction, and robust average rereferencing. The data was filtered between 0.5 and 40 Hz. An independent component analysis with automatic component classification was applied to correct for ocular and muscle artifacts. For full details, please refer to the Supplemental Material.

### Time–Frequency Decomposition, EEG-Beamforming, and Directed Connectivity Analysis

The EEG data were segmented using Brain Vision Analyzer (Version 2.1, Brain Products GmBH, Gliching, Germany) into epochs of −2000 to 4000 ms relative to the onset of the cue stimulus as well as −2000 to 4000 ms relative to the response/action effect onset. Further analysis was performed with the FieldTrip Toolbox^33^ in Matlab. TF decomposition was used to apply Morlet wavelets (number of wavelet cycles: 5; wavelets lengths: 3). Relative power changes were computed by subtracting the mean power of the baseline interval from −350 to −150 ms (relative to cue onset) from each power value, and dividing by the mean baseline power.^9^

Following Mayer et al.,^10^ 3 different successive time intervals were defined for further analysis: The “action planning” phase from 0 to 1000 ms (cue-locked), the “standby” phase from 1000 to 2000 ms (cue-locked), and the “perception” phase from 0 to 1000 ms (action–effect-locked). Each phase is assumed to be associated with specific cognitive processes. The action planning phase spanned from 0 to 1000 ms relative to cue onset. The “standby” phase covered the period from 1000 to 2000 ms relative to cue onset. The perception phase began with the onset of the action effect and lasted from 0 to 1000 ms action–effect-locked. For full details, refer to the Supplemental Material. To localize sources of action–effect associations, we reconstructed source activity using a dynamic imaging of coherent sources DICS beamforming^34^ for each phase and group. A forward model based on the Montreal Neurological Institute brain template^35^ was used to map the localized brain activity. The DICS results were thresholded by selecting voxels representing the top 3% of source activity values. For each phase (action planning, standby, perception) and group (Placebo, MPH), the selected voxels were manually grouped into regions of interest (ROIs) based on anatomical regions. Anatomical regions sharing similar functional cognitive roles were consolidated into an ROI, yielding 3 functionally dissociable ROIs for consecutive connectivity analysis. Virtual sensor time series were calculated for all selected voxels within each ROI by using the Linearly Constrained Minimum Variance (LCMV) beamformer.^36^ These time series were obtained by applying the LCMV spatial filter to the time-domain data. Finally, TF analysis was performed on the resulting time series using Morlet wavelets at a frequency of 4.5 Hz. Power values were averaged across voxels separately for each phase, group, and ROI. For full details, refer to the Supplemental Material. To assess network connectivity profiles for each phase (action planning, standby, perception, and each group [MPH/Placebo]), we applied nCREANN^30^ to capture linear and nonlinear dynamics of information flow among brain regions. nCREANN separates the linear and nonlinear connectivity by decomposing the output of the artificial neural network into 2 distinct components based on the Taylor expansion of the network’s hidden layer’s activation function. The linear part captures the linear effect of inputs on the outputs (linear connectivity), while the nonlinear part consists of higher-order terms from the Taylor series, representing nonlinear interactions between inputs and outputs. For full details, please refer to the Supplemental Material. nCREANN calculates unidirectional linear and nonlinear connectivity values for pairs of time-series data. In practice, the virtual sensor time-series data for each ROI was fed into nCREANN. The output of nCREANN is linear and nonlinear connectivity values for each possible connection of a single ROI to another ROI; in other words, an estimate of directed communication from each single brain region to another. The significance of the connectivity values was evaluated using a randomization test, involving the generation of 100 datasets with the circular time-shift surrogate method.^37^ The data were divided into consecutive time windows, each spanning half a cycle of the lowest frequency in the data (4.5 Hz). Within each segment, a random circular shift was applied to each time series, preserving the local statistical properties (mean, variance, and autocorrelation) while minimizing discontinuities at segment edges. For each connection, the estimated connectivity from the original data was compared to the null distribution of values derived from the 100 surrogate datasets. If the actual connectivity value significantly exceeds the 95th percentile of surrogate-based values, it suggests that the observed connectivity value is unlikely to be due to chance. The network configurations used for the original and surrogate data were identical.

### Statistical Analysis

A paired-sample *t*-test was used to compare the behavioral performance (mean RTs and accuracy) between the Placebo and the MPH condition (alpha = .05). The relative power changes for each datapoint were compared by means of paired-sample *t*-tests. To account for multiple testing, false discovery rate (FDR) correction was applied.^38^ To assess differences in connectivity values between ROIs, a paired-sample *t*-test was conducted for each pair, with FDR correction applied to account for multiple comparisons.^38^

## RESULTS

### Behavioral Data

As indicated by a paired-sample *t*-test (*t*(53) = -3.902; *P* < .001; *d* = 0.531), the mean reaction time (RT) of the Placebo condition (293.24 ± 7.03 ms) was significantly slower than the mean RT of the MPH condition (276.69 ± 6.51) ms. The number of correct responses did not differ between the Placebo condition (0.97 ± 0) and the MPH condition (0.98 ± 0), as revealed by a paired-sample *t*-test (*t*(53) = 1.735; *P* = .089; *d* = 0.236).

### Neurophysiological Data

**Figure 1.**
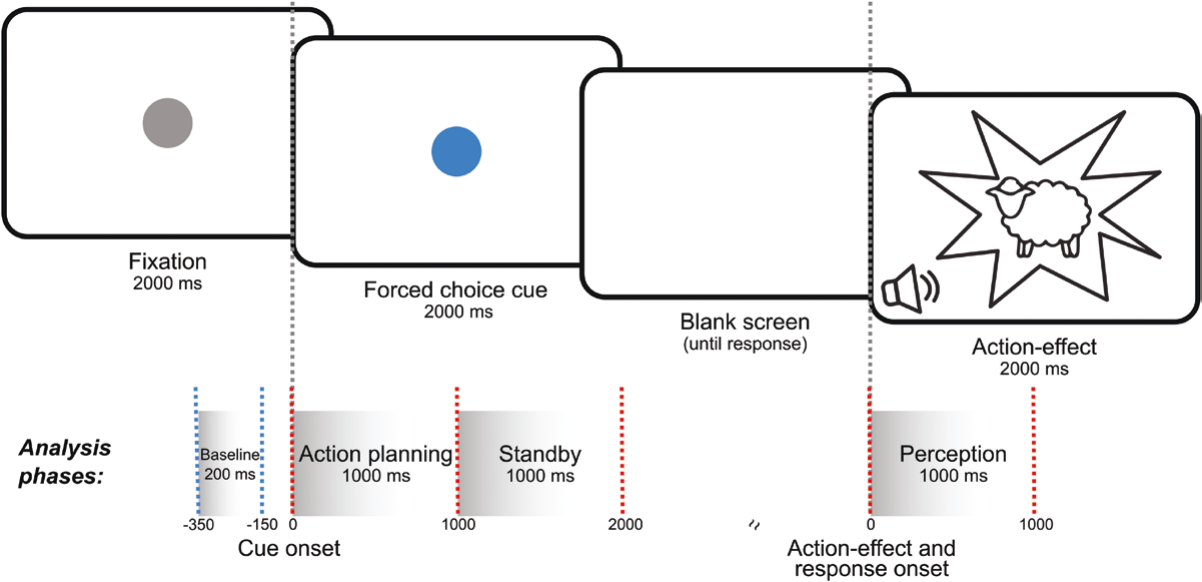
Time–frequency results. Time–frequency power of the action–effect flicker frequency at 4.5 and 8 Hz for Placebo (left) and MPH conditions (right). Averaged power values of electrodes Oz, O1, and O2 are presented. The time window is 0–2.3 seconds relative to the onset of the cue stimulus as well as −0.3 to 2 seconds relative to the action–effect onset (indicated by white dotted line). The topography plots show the power distribution at the respective action–effect frequency averaged from 0 to 2 seconds relative to action–effect onset (red dashed box).

Figure 1 shows the mean power modulations during the time course of the experiment for an action–effect flicker frequency of 4.5 and 8 Hz, separately for Placebo and MPH conditions.

**Figure 2.**
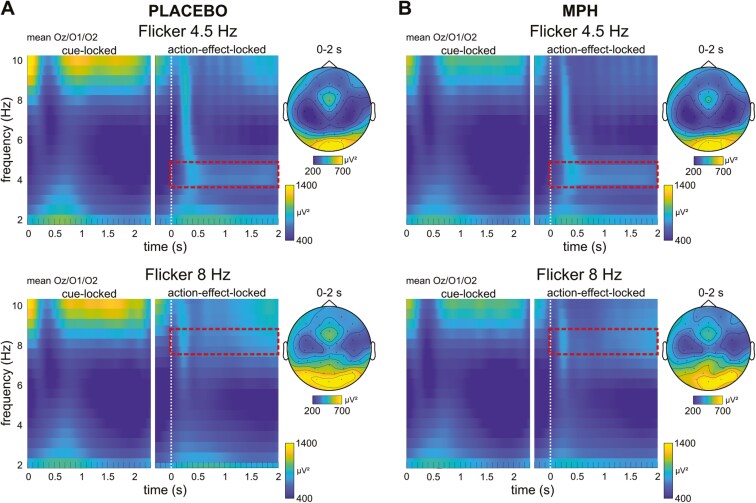
Relative power changes. Results of *t*-tests comparing relative power changes at 4.5 and 8 Hz (ie, measured frequencies) at a flicker frequency 4.5 Hz, for the Placebo (top) and the MPH conditions (bottom). For cue-locked data, all time points between 0 and 2.3 seconds were compared, for action–effect-locked data, the time window was −0.3 to 2 seconds. *P*-values (left) and *t*-values (right) are depicted by colors, nonsignificant *P*-values after false discovery rate (FDR) correction are indicated by black areas. The data were segmented into 3 different analysis phases: The “action planning” and the “standby” phases relative to the cue stimulus onset, and the “perception phase” relative to the action–effect onset.

**Figure 3.**
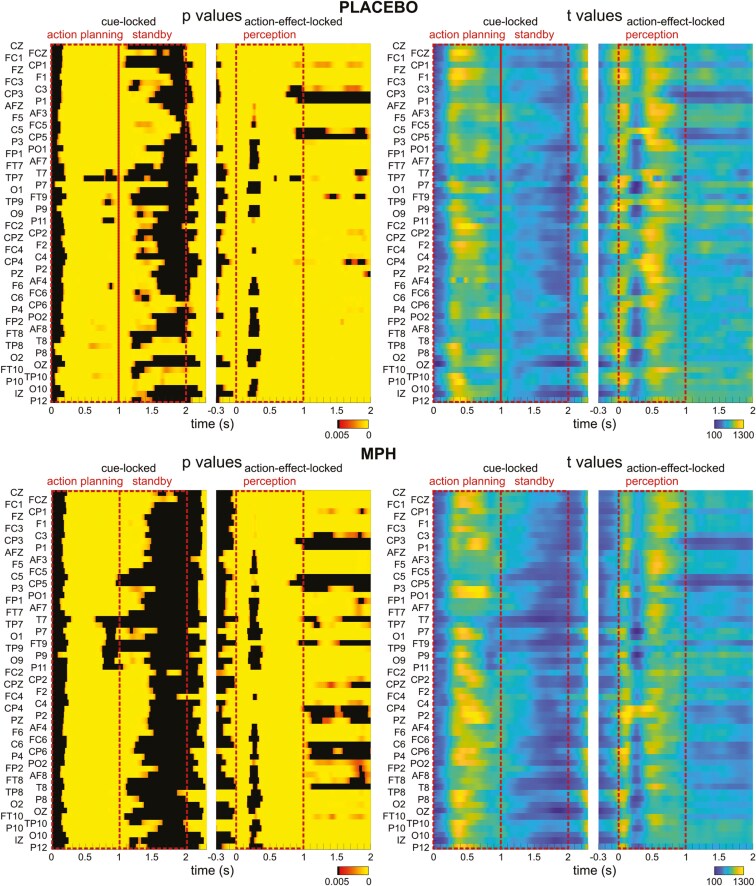
Experimental setup. Schematic depiction of the action–effect binding experiment. The timing of all stimuli is depicted. Green dashed lines indicate the baseline interval relative to the onset of the cue stimulus. Red dashed lines show the time interval of 3 analysis time windows, either relative to the onset of the cue (“action planning” phase, “standby” phase) or to the action effect (“perception” phase). The scaling of the time axis is not proportional.

Following the analysis approach of Mayer et al.,^10^ we focused on the 4.5 Hz flicker-frequency data. For the Placebo and the MPH condition, relative power changes at averaged electrodes Oz, O1, and O2 were compared between 4.5 and 8 Hz. Paired-sample *t*-tests were calculated for each data point and for each electrode (see Figure 2). For the cue-locked data, this was conducted for all time points between 0 and 2300 ms relative to the onset of the cue stimulus. For the action–effect-locked data, this was conducted between −300 and 2000 ms relative to the onset of the action effect. To account for the issue of multiple testing, FDR correction was applied.^38^ A visual inspection of the significant time points (all significant *P*-values ≤.004; *t* ≥ 3) revealed that there were significant power changes for all electrodes starting at approximately 150 ms relative to the onset to the onset of the cue stimulus (ie, cue-locked segmentation). These effects persist on almost all electrodes until about 1100 ms after which they begin to gradually diminish until about 1500 ms. The least significant time points are visible in the time window between approximately 1100 and 2000 ms, which marks the offset of the cue stimulus. In the action–effect-locked segmentation, significant time points were observed at all electrodes, commencing at the onset of the action effect/response (ie, time point 0 ms) and continuing until the end of the action effect 2000 ms later. This time interval corresponds to the time interval of the action–effect visual flicker. For further analysis, the time course was divided into 3e successive time intervals of 1000 ms each (depicted in Figures 2 and 3), as outlined in the METHODS section.

**Figure 4.**
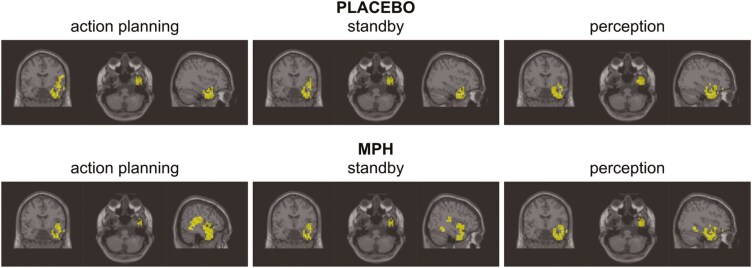
DICS source localization. Results of the DICS beamformer at 4.5 Hz and action effect of 4.5 Hz, separately for Placebo (top) and MPH condition (bottom). The beamformer revealed right-hemisphere sources in temporal, inferior frontal areas, and insular areas.

A Dynamic Imaging of Coherent Sources (DICS) beamformer was employed to reveal the cortical sources responsible for generating the theta-band activity at 4.5 Hz, separately for each analysis phase, as detailed in the METHODS section. For each phase, the top 3% of voxels contributing most to theta-band activity were identified, and the corresponding anatomical label was determined. All voxels located either outside the brain or within the white matter were excluded. Furthermore, all voxels within the cerebellar structures were removed. A DBSCAN clustering algorithm was applied to cluster the remaining voxels. All clustered voxels were labeled using the Automated Anatomical Labeling atlas version 3.^39^ The clustered voxels are illustrated in Figure 4, for details, including anatomical labels, please refer to the Table S1. Next, the voxels were manually segmented into 3 distinct functional ROIs, comprising right-hemisphere sources in the ATL, IFC, and IC areas. Anatomical regions sharing similar functional roles were consolidated into the same ROI. Individual voxels that were not assigned to any clusters were excluded. Furthermore, all voxels within subcortical nuclear structures, such as the basal ganglia, were excluded due to the inherent limitations of EEG source estimation, particularly for smaller and deeper brain structures.

### Linear and Nonlinear Effective Connectivity

To determine the linear and nonlinear effective connectivity between the reconstructed sources, nCREANN analysis was conducted.^40-42^ The models were properly trained as indicated by the model evaluation scores mean square error (MSE) and determination coefficient (*R*²): Training and testing errors were small as shown by an MSE of 0.05 (±0.03) for training data and of 0.06 ± 0.04 for testing data. Additionally, the *R*² values indicate a high fit of the model, with a mean *R*² of 0.9 ± 0.2 for training and an *R*² of 0.92 ± 0.16 for testing. The *R*² values of the training and testing phase were highly positively correlated (*r* > 0.94; *P* < .001).

**Figure 5.**
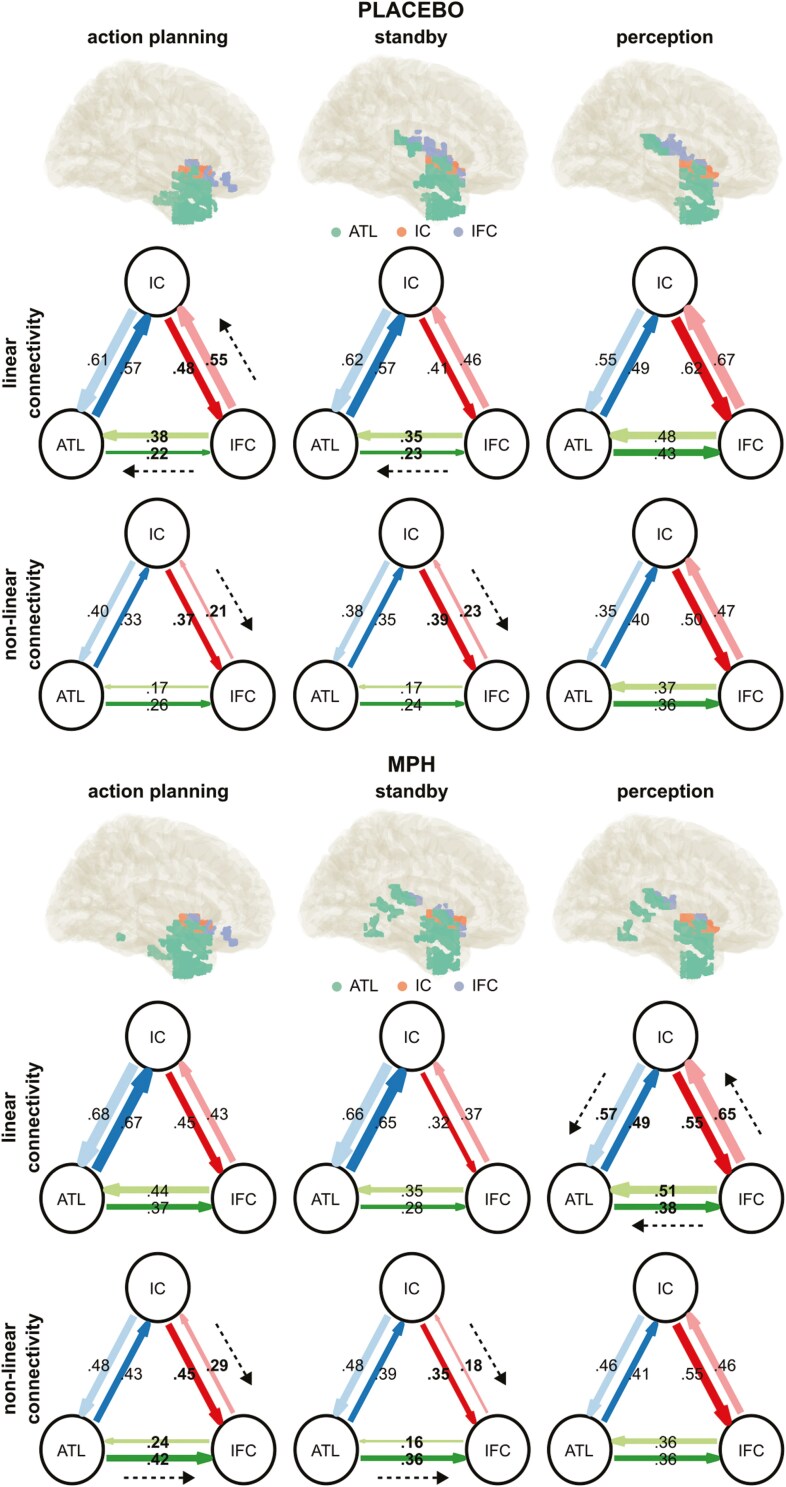
Connectivities. Linear and nonlinear effective connectivity pattern as revealed by nCREANN, for the Placebo (top) and for the MPH (bottom) condition. For each phase, linear and nonlinear connectivity pattern between established ROIs (ATL: anterior temporal lobe; IC: insular cortex (IC); IFC: inferior frontal cortex) are depicted. The direction of input flows between 2 regions of interest (ROIs) is shown by colored arrows. The connectivity strengths are depicted by the thickness of the arrows. Significant differences between bidirectional connectivities after false discovery rate (FDR) correction are indicated by bold text. Additionally, black, dashed arrows indicate significant asymmetric connectivities.

Detailed results of the nCREANN analysis are given in Table 1, including the results of paired-sample frequentist and Bayesian *t*-test comparing the bilateral connectivities. Additionally, Figure 5 shows the linear and nonlinear connectivities for Placebo and MPH conditions as well as all 3 phases. The thickness of the arrows between anatomical regions depicts the strength of the connectivity. Significant differences of bilateral connectivities are indicated by bold text (*P* < .05). FDR correction has been applied to account for multiple testing.

**Table 1.** Linear and nonlinear connectivity and results of frequentist and Bayesian *t*-test.

Phase	Connection	ROI a → b	ROI b → a	*t*	*d*	*p* (FDR)	BF10	BF evidence
Linear placebo								
Planning	ATL <> IC	0.57 ± 0.03	0.61 ± 0.04	−1.43	0.20	.189	0.39	anecdotal H0
Planning	ATL <> IFC	0.22 ± 0.02	0.38 ± 0.03	−5.73	0.79	.000	30 727.69	extreme H1
Planning	IC <> IFC	0.48 ± 0.03	0.55 ± 0.03	−3.50	0.48	.006	28.86	strong H1
Standby	ATL <> IC	0.57 ± 0.03	0.62 ± 0.04	−2.08	0.29	.086	1.08	anecdotal H1
Standby	ATL <> IFC	0.23 ± 0.02	0.35 ± 0.02	−4.01	0.55	.002	123.45	extreme H1
Standby	IC <> IFC	0.41 ± 0.03	0.46 ± 0.03	−1.55	0.21	.176	0.46	anecdotal H0
Perception	ATL <> IC	0.49 ± 0.03	0.55 ± 0.04	−2.02	0.28	.087	0.98	anecdotal H0
Perception	ATL <> IFC	0.43 ± 0.03	0.49 ± 0.04	−1.51	0.21	.177	0.43	anecdotal H0
Perception	IC <> IFC	0.62 ± 0.03	0.67 ± 0.03	−1.74	0.24	.143	0.61	anecdotal H0
Linear verum								
Planning	ATL <> IC	0.67 ± 0.03	0.68 ± 0.03	−0.19	0.03	.853	0.15	moderate H0
Planning	ATL <> IFC	0.37 ± 0.03	0.44 ± 0.03	−2.12	0.29	.086	1.16	anecdotal H1
Planning	IC <> IFC	0.45 ± 0.03	0.43 ± 0.04	0.51	0.07	.691	0.17	moderate H0
Standby	ATL <> IC	0.65 ± 0.03	0.66 ± 0.03	−0.41	0.06	.723	0.16	moderate H0
Standby	ATL <> IFC	0.28 ± 0.02	0.35 ± 0.03	−2.20	0.30	.082	1.37	anecdotal H1
Standby	IC <> IFC	0.32 ± 0.03	0.37 ± 0.03	−1.57	0.22	.176	0.47	anecdotal H0
Perception	ATL <> IC	0.49 ± 0.03	0.57 ± 0.03	−2.53	0.35	.044	2.66	anecdotal H1
Perception	ATL <> IFC	0.38 ± 0.02	0.51 ± 0.03	−3.41	0.47	.006	22.64	strong H1
Perception	IC <> IFC	0.55 ± 0.03	0.65 ± 0.04	−2.96	0.41	.017	7.23	moderate H1
Nonlinear placebo								
Planning	ATL <> IC	0.33 ± 0.03	0.40 ± 0.03	−1.73	0.24	.146	0.6	anecdotal H0
Planning	ATL <> IFC	0.26 ± 0.03	0.17 ± 0.02	2.31	0.32	.057	1.7	anecdotal H1
Planning	IC <> IFC	0.37 ± 0.03	0.21 ± 0.02	4.84	0.67	.000	1615.28	extreme H1
Standby	ATL <> IC	0.35 ± 0.03	0.38 ± 0.03	−0.69	0.09	.558	0.19	moderate H0
Standby	ATL <> IFC	0.24 ± 0.02	0.17 ± 0.02	2.21	0.30	.057	1.39	anecdotal H1
Standby	IC <> IFC	0.39 ± 0.03	0.23 ± 0.03	3.77	0.52	.001	61.87	very strong H1
Perception	ATL <> IC	0.40 ± 0.04	0.35 ± 0.03	1.14	0.16	.335	0.28	moderate H0
Perception	ATL <> IFC	0.36 ± 0.03	0.37 ± 0.03	−0.30	0.04	.807	0.16	moderate H0
Perception	IC <> IFC	0.50 ± 0.03	0.47 ± 0.04	0.85	0.12	.478	0.21	moderate H0
Nonlinear verum								
Planning	ATL <> IC	0.43 ± 0.03	0.48 ± 0.04	−1.24	0.17	.311	0.31	moderate H0
Planning	ATL <> IFC	0.42 ± 0.03	0.24 ± 0.02	5.23	0.72	.000	5724.84	extreme H1
Planning	IC <> IFC	0.45 ± 0.04	0.29 ± 0.03	3.46	0.48	.003	26.12	strong H1
Standby	ATL <> IC	0.39 ± 0.03	0.48 ± 0.04	−2.21	0.30	.057	1.4	anecdotal H1
Standby	ATL <> IFC	0.36 ± 0.03	0.16 ± 0.02	5.86	0.81	.000	47 935.7	extreme H1
Standby	IC <> IFC	0.35 ± 0.03	0.18 ± 0.02	5.47	0.75	.000	12 635.6	extreme H1
Perception	ATL <> IC	0.41 ± 0.04	0.46 ± 0.03	−1.23	0.17	.311	0.3	moderate H0
Perception	ATL <> IFC	0.36 ± 0.03	0.36 ± 0.03	0.07	0.01	.948	0.15	moderate H0
Perception	IC <> IFC	0.55 ± 0.03	0.46 ± 0.03	2.22	0.31	.057	1.43	anecdotal H1

Abbreviations: ATL, anterior temporal lobe; IC, insular cortex; IFC, inferior frontal cortex; FDR, false discovery rate; ROI, region of interest; SEM, standard error of the mean.

Mean and SEM are given for each connection beside *t*-value (*t*), Cohen’s *d* (*d*), *P*-value (*P*), and FDR-corrected *P*-value (*P* (FDR)). The Bayes factor strength of evidence label is given as evidence towards H1 if BF10 >1 and otherwise as evidence toward H0.

All linear connectivities ranged from 0.21 to 0.67 with a mean of 0.45 ± 0.03. Starting with the action planning phase of the placebo condition, the linear connectivity of ATL to IFC was significantly larger (*P* < .001) than the connectivity of IFC to ATL. Additionally, the connectivity of IC to IFC was significantly smaller than IFC to IC. There were no significant differences for ATL to IC and IC to ATL. In the placebo condition planning phase, significant differences in bilateral connectivities were only observed between ATL to IFC and IFC to ATL, with higher connectivities in the latter. No differences were found between ATL to IC and IC to ATL, as well as between IC to IFC and IFC to IC. No significant differences after FDR correction were observed for the Placebo standby phase.

For the MPH condition, the linear connectivities in the planning phase did not differ between the involved regions. Also, no differences in linear connectivities were observed in the standby phase. A different pattern emerged in the perception phase of the MPH condition. Here, the strength of linear connectivity was significantly smaller for ATL to IC (0.49) compared to IC to AFC. The linear connectivity of ATL to IFC was significantly smaller than for IFC to ATL. Additionally, a significantly smaller connectivity strength was also observed for IC to IFC in comparison to IFC to IC.

The nonlinear connectivities ranged from 0.24 to 0.55, with a mean of 0.38. In the planning phase of the placebo condition, the nonlinear connectivity of ATL to IFC was significantly larger than the connectivity of IFC to ATL. Additionally, IC to IFC (0.37) also showed a significantly larger connectivity compared to IFC to IC. No significant differences were found between ATL to IC and IC to ATL. In the standby phase of the placebo condition, the nonlinear connectivity of IC to IFC was significantly larger than that of IFC to IC. No significant differences were observed for ATL to IC and IC to ATL, as well as for ATL to IFC and IFC to ATL.

In the perception phase of the placebo condition, no significant differences were found between all involved regions. In the planning phase, the MPH condition showed several significant bilateral nonlinear connectivity differences: The connectivity strength of ATL to IFC was significantly larger than for IFC to ATL. Additionally, IC to IFC showed a larger connectivity value compared to IFC to IC. No differences were observed for ATL to IC compared to IC to ATL. In the standby phase of the MPH condition, all bilateral connectivities differed significantly. Connectivities were smaller for ATL to IC compared to IC to ATL, but larger for ATL to IFC compared to IFC to ATL. Significantly stronger nonlinear connectivities were also observed for IC to IFC compared to IFC to IC. Finally, in the perception phase of the MPH condition, no significant difference in bilateral nonlinear connectivities was observed.

## DISCUSSION

We focused on the role of the catecholaminergic system as a possible modulator of directed communication in cortical theta-band activity networks during AEB processes. According to ideomotor principles 1 and 2, AEBs constitute the building blocks of intentional behavior; thus, the study provides mechanistic insights into a core aspect of action control. The neurophysiological findings, particularly of the EEG-beamforming analysis, replicate 2 aspects of our previous findings^10^ in an independent sample: First, theta-band activity in the IC, ATL, and IFC is involved in different stages of the AEB dynamic.^10^ Second, a bidirectional information transfer between these regions in the planning, standby, and perception phases takes place.

Interestingly, the pharmacological intervention resulted in distinct patterns of connectivity involved in AEBs, indicating condition-specific changes in the directionality of cortical neural communication. These findings line up with notions that gain modulation and its effect on cognitive functions can also affect how information is routed through different network parts,^24-26^ and can affect directed connectivity in a network.^28,29^ More specifically, we found that the asymmetrical communication between IC, ATL, and IFC was modulated by the administration of the MPH. In the placebo condition, the information transfer (linear connectivity) was significantly stronger from the IFC to the ATL than vice versa. This was the case for both the planning and standby phases; however, nonlinear connectivity revealed a significant effect of the ATL on the IFC during the planning phase. Importantly, this pattern consistently changed when MPH was administered. After administering MPH, information transfer to the IFC was evident, and this is the case for the ATL–IFC connection and the IC–IFC connection. This suggests that IFC became a “hub” in the planning and standby phases.

During the planning phase of an action, predictions of the precise effect of the action must be made and maintained during the standby phase.^10^ For both, the prediction of the action effects and their maintenance, theta-band activity is important.^12,13,43-46^ The IC subserves sensorimotor integration^47-51^ and the ATL distills coherent concepts from multimodal inputs.^10,52^ The ATL likely needs sensorimotor information from the IC to distill this information into a coherent representation.^10^ This is substantiated by the bidirectional transfer of information found in the placebo and the MPH condition in the planning and the standby phase. The observed transfer of information from the ATL and IC to the IFC during the planning and standby phases under MPH suggests a more active engagement of the IFC with this information in these phases when catecholaminergic activity is increased. As argued previously,^10^ the IFC serves many different cognitive functions,^53-55^ and especially information transfer between ATL and IFC serves (working) memory processes.^56-58^ Due to the design of the action–effect experiment, the action effect implied by the action plan (in the planning phase) is reactivated by the possible onset of the self-generated action effect in the perception phase.

In tasks that require evaluating action outcomes, the right IFC plays a central role in assessing the success of actions and assigning value to their effects. Information from the IC and ATL is critical for this evaluation, making it plausible that under MPH, IFC has enhanced access to this information through altered network dynamics. Indeed, MPH modulated the directed connectivity during the perception phase, reversing the pattern observed in the planning and standby phases. Specifically, information flow shifted from the IFC to the IC and ATL, suggesting that once the action effect becomes perceptually evident, evaluative processing in the IFC is reduced, and information is redistributed to support downstream integration. This aligns with findings highlighting the interplay between ATL and IFC in retrieving and evaluating perceptual-motor representations.^10,15,59^ Thus, MPH likely modulates which of the involved brain structures acts as a “hub” in different phases of the action effect binding process. Notably, the ATL emerged as the primary recipient of information during the perception phase, indicating a shift in network hub status from IFC (in planning and standby) to ATL (in perception). This may reflect the ATL’s role in consolidating sensorimotor input from IC^10^ with evaluative feedback from IFC to update action–effect associations.

Crucially, MPH administration not only modulated the asymmetry of directed communication within the theta band but also altered the organization of this communication—specifically, the relevance of linear and nonlinear information flow. The applied method to estimate directed communication distinguishes between linear and nonlinear directed communication,^30^ a distinction shown to be particularly relevant for action control processes.^13,40,41^ This is likely due to the complex interplay of feedforward and feedback loops in perception–action integration, in which linear and nonlinear dynamics play a role.^60-62^ Indeed, action–effect integration was dependent on linear and nonlinear directed communication, with both types of communication evident across all phases of the experiment. This replicates previous findings.^10^ Especially nonlinear directed communication facilitated directed information transfer from the ATL to the IFC during the planning and standby phases after MPH administration. Since MPH is thought to increase gain modulation and how information is routed through different network parts,^24-26^ this suggests that nonlinear communication allows more efficient or flexible network communication for action preparation and control. This interpretation aligns with studies suggesting that nonlinear information transfer is more efficient than linear information transfer during action control. However, in the perception phase, the MPH-induced change in the asymmetry of the directed transfer of information from the IFC and the IC to the ATL was mainly linear.

Importantly, the linear and nonlinear directed connectivity analyses offer important insights since they revealed complex dynamics critical for adaptive cognitive control.^63^ The current findings suggest a dynamic reorganization of communication modes across task phases, with nonlinear interactions supporting flexible planning and action control during the preparatory stages, while linear communication becomes more prominent during the evaluation of sensory outcomes. This shift in communication dynamics underscores the functional relevance of distinguishing between linear and nonlinear directed information transfer patterns. The catecholaminergic system alters directed communication within cortical theta-band activity, enhancing information flow during AEB. Catecholamines optimize network-level processing supporting goal-directed behavior and action regulation. The study’s findings highlight how the neural organization of processes supporting intentional action can be optimized neurobiologically.

### Limitation and Outlook

Although the findings underscore the potential of pharmacological interventions to shape action–effect learning electrophysiologically, future studies may combine EEG with fMRI to pinpoint also subcortical loci of catecholaminergic modulation. This is relevant since the catecholaminergic system originates from subcortical nuclei, and the basal ganglia also play an important role in action selection. This may then also include analyses of cross-frequency interactions, possibly central for event file coding.^12^ Additionally, more selective pharmacological approaches targeting specific receptor system will provide fine-grained insights into the neurobiology of AEB. In that regard, animal experimental studies will be relevant since these allow a fine-grained assessment of receptor systems through the specific receptor antagonists. This will also broaden the scope of action–effect learning, which is relevant for any species. These studies may then also consider sex differences in the form of steroid hormone–neurotransmitter interactions, also using steroid hormone administration.

## Supplementary Material

Supplementary material are available at *International Journal of Neuropsychopharmacology* (IJNPPY) online.

pyaf031_suppl_Supplementary_Table_S1

## Data Availability

The data underlying this article will be shared on reasonable request to the corresponding author.
