# Supplementary material for: Pharmacological modulation of directed network communication and neural hubs in action–effect integration
Source: Int J Neuropsychopharmacol. 2025 May 27;28(6):pyaf031. doi: 10.1093/ijnp/pyaf031 (PMC12143126; doi:10.1093/ijnp/pyaf031)
Supplement: pyaf031_suppl_Supplementary_Table_S1 [file pyaf031_suppl_supplementary_table_s1.docx]

**Supplemental Material**

**Pharmacological modulation of directed network communication and neural hubs in action-effect integration**

Jasmin Mayer, Anna Helin Koyun, Moritz Mückschel, Veit Roessner, Bernhard Hommel, Christian Beste

*EEG recording and pre-processing*

EEG recordings were done with QuickAmp and BrainAmp amplifiers (Brain Products GmbH, Gilching, Germany), applying 60 Ag-AgCl electrodes in an equidistant setup with a 500 Hz sampling rate (reference electrode at Fpz, impedances < 5 kΩ). Preprocessing was done in automagic ^1^ using EEGLAB ^2^ and Matlab (2021b, The MathWorks Corp). The EEG data was downsampled to 256 Hz and flat EOG channels were interpolated. We subsequently applied the PREP preprocessing pipeline ^3^, which applied a multitaper algorithm to eliminate 50 Hz line noise and implements a resilient average reference following the removal of artifacts from bad channels. We applied EEGLAB clean_rawdata() pipeline to detrend the EEG data (FIR high-pass filter of 0.5 Hz, order 1286, stop-band attenuation 80 dB, transition band 0.25 – 0.75 Hz). Flat lines and channels consisting of outliers or noise were identified and removed. Next, epochs with arbitrary high power (>15 standard deviations compared to calibration data) were reconstructed utilizing Artifact Subspace Reconstruction (ASR; burst criterion: 15;^4^). Time windows that could not be reconstructed were excluded. The EEG data was further lowpass filtered at 40 Hz using a sinc FIR filter with an order of 86 (^5^. Eye movement artifacts were mitigated using the Parra et al. ^6^ subtraction method. Independent Component Analysis (ICA) and the Multiple Artifact Rejection Algorithm (MARA; ^7,8^) were applied to remove remaining muscle and eye artifacts and loose electrodes. ICLabel (threshold: 0.8; ^9^) identified and removed cardiac artifacts. Finally, we applied a spherical method to interpolate all removed channels.

*Time-frequency decomposition*

The EEG data was segmented using Brain Vision Analyzer (Version 2.1, Brain Products GmBH, Gliching, Germany) into epochs of -2000 to 4000 ms relative to the onset of the cue stimulus as well as -2000 to 4000 ms relative to the response/action effect onset. Further analysis was performed with the FieldTrip Toolbox ^10^ in Matlab. Time-frequency (TF) decomposition was used to apply Morlet wavelets (number of wavelet cycles: 5; wavelets lengths: 3). Relative power changes were computed by subtracting the mean power of the baseline interval from -350 ms to -150 ms (relative to cue onset) from each power value, and dividing by the mean baseline power ^11^.

Following Mayer et al. ^12^, three different successive time intervals were defined for further analysis: The “action planning” phase from 0 ms to 1000 ms (cue-locked), the “standby” phase from 1000 to 2000 ms (cue-locked), and the “perception” phase from 0 to 1000 ms (action-effect-locked). Each phase is assumed to be associated with specific cognitive processes. The action planning phase spanned from 0 to 1000 ms relative to cue onset. This phase involves processing the cue stimulus and selecting the appropriate response, characterized by the N1-N2-P3 event-related potential complex ^e.g. 13–16^ and theta band activity ^17,18^ within the first 600 ms. To allow for four cycles of 4.5 Hz oscillations, ensuring robust frequency resolution, the phase was prolonged to 1000 ms. The “standby” phase covered the period from 1000 to 2000 ms relative to cue onset. By this stage, response selection is assumed to be complete, and the response execution remains on hold until the go signal.

The perception phase began with the onset of the action effect and lasted from 0 to 1000 ms action-effect-locked. This phase includes the perceptual processing of the action effect, the binding of perceptual features, and post-response evaluation ^e.g. 19–21^. The lengths of all three phases were kept the same to ensure comparability and avoid interference from differing sample point counts.

*DICS beamformer*

To localize sources of action-effect associations, we reconstructed the source activity using a dynamic imaging of coherent sources DICS beamforming ^22^ for each phase (action-planning, standby, perception) and substance group (Placebo, MPH). A forward model based on the Montreal Neurological Institute (MNI) brain template ^23^ was used to map the localized brain activity from the DICS beamformer onto a gridded source space using FieldTrip ^10^. Spectral analysis was performed using a Hanning taper to calculate power values and the cross-spectral density matrix within the frequency range of 4 to 5 Hz. A leadfield matrix was calculated by dividing the brain volume into a grid with a 5 mm resolution. For all conditions a common spatial filter was applied (regularization parameter set at 5%). To correct for noise bias towards the center of the head, we normalized the source estimation by an estimate of spatially inhomogeneous noise for each voxel ^24^, based on the smallest eigenvalue of the cross-spectral density matrix.

*Selection of regions of interest (ROI) and LCMV beamformer*

Regions of interest (ROI) for the consecutive nCREANN analysis were defined based on the results of the DICS beamformer. Voxels located outside the brain, within the cerebellum, and in white matter were excluded. Voxels representing the top 3% of source activity values were then selected. These voxels were clustered using the Density-Based Spatial Clustering of Applications with Noise (DBSCAN) algorithm ^25,26^, which identifies clusters of irregular shapes in dispersed data without requiring a predetermined cluster count. An epsilon value equivalent to twice the edge length of each voxel was chosen to ensure detection of neighboring voxels. Using the resulting clusters and labels from the Automated Anatomical Labeling (AAL) atlas version 3 ^27^, regions of interest (ROIs) were manually identified for each phase (action planning, standby, and perception). Anatomical regions sharing similar functional roles were consolidated into the same ROI. Table S1 lists the anatomical regions and selected number of voxels for each phase and ROI. Virtual sensor time series were calculated for all voxels within each ROI and phase using the Linearly Constrained Minimum Variance (LCMV) beamformer ^24^. These time series were obtained by applying the LCMV spatial filter to the time-domain data. Finally, time-frequency (TF) analysis was performed on the resulting time series using Morlet wavelets at a frequency of 4.5 Hz. Power values were averaged across voxels separately for each phase, group, and ROI.

*Table S1: Region of interest (ROI) definition: Number of voxels per anatomical region and assigned ROI after DICS beamformer and DBSCAN clustering. The respective hemisphere for each anatomical region is given in brackets: right (R) or left (L).Voxels listed under “Excluded”*

| **ROI** | **Anatomical regions** | **Placebo** | | | **MPH** | | |
| --- | --- | --- | --- | --- | --- | --- | --- |
|  |  | **action planning** | **standby** | **perception** | **action planning** | **standby** | **perception** |
| ATL | Temporal pole: superior temporal gyrus (R) | 49 | 44 | 40 | 41 | 37 | 30 |
|  | Parahippocampal gyrus (R) | 32 | 9 | 8 | 23 | 2 | 3 |
|  | Inferior temporal gyrus (R) | 36 | 33 | 33 | 40 | 42 | 47 |
|  | Middle temporal gyrus (R) | 11 | 20 | 11 | 29 | 41 | 29 |
|  | Temporal pole: middle temporal gyrus (R) | 47 | 40 | 39 | 35 | 23 | 24 |
|  | Superior temporal gyrus (R) | 7 | 31 | 37 | 18 | 71 | 64 |
| IC | Insula (R) | 38 | 30 | 32 | 34 | 30 | 27 |
| IFC | Inferior frontal gyrus, opercular part (R) | 1 | 1 | 1 | 1 | 1 | 0 |
|  | Inferior frontal gyrus, pars orbitalis (R) | 20 | 3 | 2 | 13 | 1 | 0 |
|  | Rolandic operculum (R) | 1 | 48 | 41 | 2 | 20 | 20 |
| Excluded | Amygdala (R) | 11 | 7 | 8 | 8 | 2 | 4 |
|  | Fusiform Gyrus (L) | 0 | 0 | 0 | 7 | 4 | 13 |
|  | Fusiform Gyrus (R) | 16 | 10 | 8 | 26 | 16 | 25 |
|  | Heschl’s gyrus (R) | 0 | 2 | 4 | 0 | 6 | 6 |
|  | Hippocampus (R) | 17 | 4 | 3 | 17 | 3 | 3 |
|  | Olfactory cortex (R) | 3 | 1 | 1 | 1 | 0 | 0 |
|  | Postcentral gyrus (R) | 0 | 9 | 6 | 0 | 0 | 0 |
|  | Putamen (R) | 7 | 0 | 1 | 3 | 0 | 0 |
|  | SupraMarginal_R gyrus (R) | 0 | 4 | 20 | 0 | 0 | 3 |

Abbreviations: ROI: regions of interest; ATL: Anterior temporal lobe; IC: Insular cortex; IFC: Inferior frontal cortex; MPH: methylphenidate

*Non-linear causal relationship estimation by artificial neural networks (nCREANN)*

To assess network connectivity profiles of both conditions (MPH/Placebo) we applied nCREANN (nonlinear Causal Relationship Estimation by Artificial Neural Network) utilizing artificial neural networks (ANNs) for the estimation of effective connectivity among multiple brain regions. nCREANN method builds upon a nonlinear Multivariate Autoregressive (MVAR) model, in which interactions of different brain regions at current time points are influenced by their prior activities and is presented by the following equation:

| $\mathbf{x}\left( n \right)=\boldsymbol{f}\left( \mathbf{x}_{p} \right)+\boldsymbol{\sigma}\left( n \right)$ | (1) |
| --- | --- |
|  |  |

with $\mathbf{x}_{p}=\left[ x_{1}\left( n-1 \right),x_{2}\left( n-1 \right), \cdots,x_{M}\left( n-p \right) \right]^{T}$denoting the vector of *p* previous samples of *M* time series and $\boldsymbol{\sigma}\left( n \right)= \left[ \sigma_{1}, \sigma_{2}, \ldots, \sigma_{M} \right]^{T}$ representing the residual of the model.

Note, that nCREANN allows to capture linear and nonlinear dynamics of information flow among brain regions, as brain interactions often exhibit high nonlinearity, and linear methods may oversimplify the intricate functions of the brain. Assessing both linear and nonlinear concepts is necessary for an inclusive understanding of brain mechanism on a macroscale ^29–33^.

In nCREANN, a single-hidden-layer feedforward neural network is used to implement the nonlinear Multivariate Autoregressive (nMVAR) model. This network incorporates nonlinear activation functions for hidden neurons and linear functions at the output layer. To evaluate both linear and nonlinear information within the network linear ${\boldsymbol{(}\boldsymbol{f}}^{Lin})$ and nonlinear $\boldsymbol{f}^{NonLin}$ parts of the network input-output mapping $\boldsymbol{f}\left( . \right)$ are separated:

| $\boldsymbol{f}=\boldsymbol{f}^{Lin}+\boldsymbol{f}^{NonLin}$ | (2) |
| --- | --- |

This separation is achieved by decomposing the hidden neurons' functions into their linear and nonlinear components using Taylor series expansion.

Linear effective connectivity ${(lC}_{i\to j})$ is calculated by multiplying the network's connection weights with the scaling parameters of the hidden neurons $\boldsymbol{f}^{Lin}$. This measure indicates the extent to which the *i*^th^ input node linearly influences the the *j*^th^ output neuron. For more detauls refer to Talebi et al. ^34^. Nonlinear effective connectivity (${NC}_{i\to j}$) from node $x_{i}$ to node $x_{j}$ is defined as the ratio of the network's estimation errors:

${NC}_{i\to j}=\ln\left( \frac{\left\langle{\left( \epsilon_{j} \right)_{x_{i}\_Lin}}^{2} \right\rangle}{\left\langle\left( \epsilon_{j} \right)^{2} \right\rangle} \right)$ (3)

In the numerator, the estimation error refers to scenarios where the influence of node $x_{i}$ on node $x_{j}$ is purely linear. In the denominator, the estimation error corresponds to situations where all input signals exert both linear and nonlinear effects on node $x_{j}$i. ${NC}_{i\to j}$ quantifies the extent of the nonlinear causal effect of node $x_{i}$ on node $x_{j}$.

The optimal model order was calculated for each condition individually using the Schwarz Bayesian Criterion (p = 12) in the ARfit toolbox (Multivariate Autoregressive Model Fitting; ^35,36^).

The Multilayer Perceptron neural network consisted of 1 hidden layer and 10 hidden neurons and was trained with the gradient descent error back-propagation (EBP) algorithm with momentum (*α*) and adaptive learning rate (*η*). Next, we applied early stopping as generalization method with a 5-fold permuted cross-validation and split the data into 80% training, 10% validation, and 10% testing sets. To update the network, we applied the ‘incremental mode’ method with random parameters initially ranging from -0.5 to 0.5. The power values for each subject were normalized to the range [0, 1.5] prior to applying nCREANN, using a scaling factor calculated as 1.5/(maximum of subject’s power values). This preprocessing step ensures that the computation of directed connectivity metrics is not influenced by individual differences in power values. The resulting connectivity patterns are represented schematically, with arrows illustrating the flow of information between clusters of sources. The thickness and size of the arrows are proportional to the strength of the connectivity. The model’s performance was assessed using the Mean Square Error (MSE) and the coefficient of determination (R²) for both training and test datasets. MSE is a commonly used metric to evaluate network performance, with well-trained networks demonstrating low training errors and test errors that fall within the range of training errors. The R² metric measures the quality of regression models, where values approaching 1 indicate a closer fit to the optimal model. Similar R² values for the training and test sets highlight the network’s effective generalization. The significance of the connectivity values was further evaluated using a randomization test, involving the generation of 100 datasets with the circular time-shift surrogate method ^37^. The data was divided into consecutive time windows, each spanning half a cycle of the lowest frequency in the data (4.5 Hz). Within each segment, a random circular shift was applied to each time series, preserving the local statistical properties (mean, variance, and autocorrelation) while minimizing discontinuities at segment edges. For each connection, the estimated connectivity from the original data was compared to the range of values derived from the surrogate data. This comparison involved calculating the 95th percentile of the surrogate connectivity distribution. The network configurations used for the original and surrogate data were identical.

***Quantification and statistical analysis***

A paired-sample t-test was used to compare the behavioral performance (mean RTs and accuracy) between the Placebo and the MPH condition (alpha = 0.05). The relative power changes for each datapoint were compared by means of paired-sample t-tests. To account for multiple testing FDR correction was applied ^38^. To assess differences in connectivity values between regions of interest (ROI), a paired-sample t-test was conducted for each pair, with false discovery rate (FDR) correction applied to account for multiple comparisons ^38^.

**References**

1. Pedroni A, Bahreini A, Langer N. Automagic: Standardized preprocessing of big EEG data. *Neuroimage*. 2019;200:460-473. doi:10.1016/j.neuroimage.2019.06.046

2. Delorme A, Makeig S. EEGLAB: an open source toolbox for analysis of single-trial EEG dynamics including independent component analysis. *Journal of Neuroscience Methods*. 2004;134(1):9-21. doi:10.1016/j.jneumeth.2003.10.009

3. Bigdely-Shamlo N, Mullen T, Kothe C, Su KM, Robbins KA. The PREP pipeline: standardized preprocessing for large-scale EEG analysis. *Frontiers in Neuroinformatics*. 2015;9. Accessed September 20, 2023. https://www.frontiersin.org/articles/10.3389/fninf.2015.00016

4. Mullen T, Kothe C, Chi YM, et al. Real-Time Modeling and 3D Visualization of Source Dynamics and Connectivity Using Wearable EEG. *Conf Proc IEEE Eng Med Biol Soc*. 2013;2013:2184-2187. doi:10.1109/EMBC.2013.6609968

5. Widmann A, Schröger E, Maess B. Digital filter design for electrophysiological data – a practical approach. *Journal of Neuroscience Methods*. 2015;250:34-46. doi:10.1016/j.jneumeth.2014.08.002

6. Parra LC, Spence CD, Gerson AD, Sajda P. Recipes for the linear analysis of EEG. *NeuroImage*. 2005;28(2):326-341. doi:10.1016/j.neuroimage.2005.05.032

7. Winkler I, Haufe S, Tangermann M. Automatic classification of artifactual ICA-components for artifact removal in EEG signals. *Behav Brain Funct*. 2011;7:30. doi:10.1186/1744-9081-7-30

8. Winkler I, Brandl S, Horn F, Waldburger E, Allefeld C, Tangermann M. Robust artifactual independent component classification for BCI practitioners. *J Neural Eng*. 2014;11(3):035013. doi:10.1088/1741-2560/11/3/035013

9. Pion-Tonachini L, Kreutz-Delgado K, Makeig S. The ICLabel dataset of electroencephalographic (EEG) independent component (IC) features. *Data in Brief*. 2019;25:104101. doi:10.1016/j.dib.2019.104101

10. Oostenveld R, Fries P, Maris E, Schoffelen JM. FieldTrip: Open Source Software for Advanced Analysis of MEG, EEG, and Invasive Electrophysiological Data. *Computational Intelligence and Neuroscience*. 2010;2011:e156869. doi:10.1155/2011/156869

11. Dignath D, Kiesel A, Frings C, Pastötter B. Electrophysiological evidence for action-effect prediction. *J Exp Psychol Gen*. 2020;149(6):1148-1155. doi:10.1037/xge0000707

12. Mayer J, Mückschel M, Talebi N, Hommel B, Beste C. Directed connectivity in theta networks supports action-effect integration. *NeuroImage*. 2025;305:120965. doi:10.1016/j.neuroimage.2024.120965

13. Folstein JR, Van Petten C. Influence of cognitive control and mismatch on the N2 component of the ERP: A review. *Psychophysiology*. 2008;45(1):152-170. doi:10.1111/j.1469-8986.2007.00602.x

14. Luck S. *An Introduction to Event-Related Potentials and Their Neural Origins (Chapter 1)*. MIT Press; 2005.

15. Näätänen R, Picton T. The N1 Wave of the Human Electric and Magnetic Response to Sound: A Review and an Analysis of the Component Structure. *Psychophysiology*. 1987;24(4):375-425. doi:10.1111/j.1469-8986.1987.tb00311.x

16. Polich J. Updating P300: An integrative theory of P3a and P3b. *Clinical Neurophysiology*. 2007;118(10):2128-2148. doi:10.1016/j.clinph.2007.04.019

17. Cavanagh JF, Frank MJ. Frontal theta as a mechanism for cognitive control. *Trends in Cognitive Sciences*. 2014;18(8):414-421. doi:10.1016/j.tics.2014.04.012

18. Nigbur R, Ivanova G, Stürmer B. Theta power as a marker for cognitive interference. *Clinical Neurophysiology*. 2011;122(11):2185-2194. doi:10.1016/j.clinph.2011.03.030

19. Danielmeier C, Ullsperger M. Post-Error Adjustments. *Front Psychol*. 2011;2. doi:10.3389/fpsyg.2011.00233

20. Falkenstein M, Hoormann J, Christ S, Hohnsbein J. ERP components on reaction errors and their functional significance: a tutorial. *Biological Psychology*. 2000;51(2):87-107. doi:10.1016/S0301-0511(99)00031-9

21. Miyake A, Friedman NP. The Nature and Organization of Individual Differences in Executive Functions: Four General Conclusions. *Curr Dir Psychol Sci*. 2012;21(1):8-14. doi:10.1177/0963721411429458

22. Gross J, Kujala J, Hämäläinen M, Timmermann L, Schnitzler A, Salmelin R. Dynamic imaging of coherent sources: Studying neural interactions in the human brain. *Proceedings of the National Academy of Sciences*. 2001;98(2):694-699. doi:10.1073/pnas.98.2.694

23. Collins DL, Zijdenbos AP, Kollokian V, et al. Design and construction of a realistic digital brain phantom. *IEEE Transactions on Medical Imaging*. 1998;17(3):463-468. doi:10.1109/42.712135

24. Van Veen BD, Van Drongelen W, Yuchtman M, Suzuki A. Localization of brain electrical activity via linearly constrained minimum variance spatial filtering. *IEEE Transactions on Biomedical Engineering*. 1997;44(9):867-880. doi:10.1109/10.623056

25. Adelhöfer N, Schreiter ML, Beste C. Cardiac cycle gated cognitive-emotional control in superior frontal cortices. *NeuroImage*. 2020;222:117275. doi:10.1016/j.neuroimage.2020.117275

26. Ester M, Kriegel HP, Sander J, Xu X. A density-based algorithm for discovering clusters in large spatial databases with noise. In: *Proceedings of the Second International Conference on Knowledge Discovery and Data Mining*. KDD’96. AAAI Press; 1996:226-231.

27. Rolls ET, Huang CC, Lin CP, Feng J, Joliot M. Automated anatomical labelling atlas 3. *NeuroImage*. 2020;206:116189. doi:10.1016/j.neuroimage.2019.116189

28. Lee MD, Wagenmakers EJ. *Bayesian Cognitive Modeling: A Practical Course*. Cambridge University Press; 2014. doi:10.1017/CBO9781139087759

29. Friston KJ. Book Review: Brain Function, Nonlinear Coupling, and Neuronal Transients. *The Neuroscientist*. 2001;7(5):406-418. doi:10.1177/107385840100700510

30. Chen CC, Henson RN, Stephan KE, Kilner JM, Friston KJ. Forward and backward connections in the brain: A DCM study of functional asymmetries. *NeuroImage*. 2009;45(2):453-462. doi:10.1016/j.neuroimage.2008.12.041

31. Ferdousi M, Babaie-Janvier T, Robinson PA. Nonlinear wave-wave interactions in the brain. *Journal of Theoretical Biology*. 2020;500:110308. doi:10.1016/j.jtbi.2020.110308

32. Nozari E, Bertolero MA, Stiso J, et al. Is the brain macroscopically linear? A system identification of resting state dynamics. Published online 2020. doi:10.48550/ARXIV.2012.12351

33. Cifre I, Miller Flores MT, Penalba L, Ochab JK, Chialvo DR. Revisiting Nonlinear Functional Brain Co-activations: Directed, Dynamic, and Delayed. *Front Neurosci*. 2021;15:700171. doi:10.3389/fnins.2021.700171

34. Talebi N, Nasrabadi AM, Mohammad-Rezazadeh I. Estimation of effective connectivity using multi-layer perceptron artificial neural network. *Cogn Neurodyn*. 2018;12(1):21-42. doi:10.1007/s11571-017-9453-1

35. Neumaier A, Schneider T. Estimation of parameters and eigenmodes of multivariate autoregressive models. *ACM Trans Math Softw*. 2001;27(1):27-57. doi:10.1145/382043.382304

36. Schneider T, Neumaier A. Algorithm 808: ARfit—a matlab package for the estimation of parameters and eigenmodes of multivariate autoregressive models. *ACM Trans Math Softw*. 2001;27(1):58-65. doi:10.1145/382043.382316

37. Papana A, Kyrtsou C, Kugiumtzis D, Diks C. Simulation Study of Direct Causality Measures in Multivariate Time Series. *Entropy*. 2013;15(7):2635-2661. doi:10.3390/e15072635

38. Genovese CR, Lazar NA, Nichols T. Thresholding of Statistical Maps in Functional Neuroimaging Using the False Discovery Rate. *NeuroImage*. 2002;15(4):870-878. doi:10.1006/nimg.2001.1037
